# Expanding health technology assessment towards broader value: Ireland as a case study

**DOI:** 10.1017/S0266462323000235

**Published:** 2023-05-02

**Authors:** Irina Kinchin, Valerie Walshe, Charles Normand, Joanna Coast, Rachel Elliott, Thilo Kroll, Philip Kinghorn, Alexander Thompson, Rosalie Viney, David Currow, James F. O’Mahony

**Affiliations:** 1 Centre for Health Policy and Management, Trinity College Dublin, The University of Dublin, Dublin, Ireland; 2 Health Service Executive, Dublin, Ireland; 3 Bristol Population Health Science Institute, University of Bristol, Bristol, UK; 4 Manchester Centre for Health Economics, The University of Manchester, Manchester, UK; 5 School of Nursing, Midwifery and Health Systems, University College Dublin, Dublin, Ireland; 6 Health Economics Unit, Institute of Applied Health Research, University of Birmingham, Birmingham, UK; 7 Centre for Health Economics Research and Evaluation (CHERE), University of Technology Sydney, Ultimo, NSW, Australia; 8 Faculty of Science, Medicine and Health, University of Wollongong, Wollongong, NSW, Australia

**Keywords:** Health technology assessment (HTA), resource allocation, outcome measurement, benefits beyond health, wellbeing, social value

## Abstract

Healthcare innovations often represent important improvements in population welfare, but at what cost, and to whom? Health technology assessment (HTA) is a multidisciplinary process to inform resource allocation. HTA is conventionally anchored on health maximization as the only relevant output of health services. If we accept the proposition that health technologies can generate value outside the healthcare system, resource allocation decisions could be suboptimal from a societal perspective. Incorporating “broader value” in HTA as derived from social values and patient experience could provide a richer evaluative space for informing resource allocation decisions. This article considers how HTA is practiced and what its current context implies for adopting “broader value” to evaluating health technologies. Methodological challenges are highlighted, as is a future research agenda. Ireland serves as an example of a healthcare system that both has an explicit role for HTA and is evolving under a current program of reform to offer universal, single-tier access to public services. There are various ways in which HTA processes could move beyond health, including considering the processes of care delivery and/or expanding the evaluative space to some broader concept of well-being. Methods to facilitate the latter exist, but their adaptation to HTA is still emerging. We recommend a multi-stakeholder working group to develop and advance an international agenda for HTA that captures welfare/benefit *beyond health.*

## Introduction

No healthcare system has enough resources to fund every clinically effective intervention. Investing in new interventions requires reallocation of funds from other services, within or outside healthcare. Health technology assessment (HTA) is a multidisciplinary process incorporating medical, social, economic, and ethical principles that can inform resource allocation decisions (1). HTA aims to combine technical and normative value judgments explicitly, thereby avoiding implicit rationing. Health technology may include any intervention as well as organizational and support systems to enhance health. Ultimately, HTA aims to inform safe, effective, equitable, and sustainable health policies (1).

Although HTA’s application is increasingly widespread, aspects of its implementation vary internationally. In particular, the application of economic evaluation within HTA, its purpose, methodological guidelines, and underpinning normative choices all vary across countries (2). This reflects differences in healthcare expenditure, funding mechanisms, and political support.

An important issue that varies between countries is the perspective on outcomes. Many HTA agencies solely focus on health maximization, assuming health ought to be the healthcare system’s primary product. Some countries seek to maximize health as measured by quality-adjusted life-years (QALYs), including Austria, Ireland, and the UK (2). QALYs facilitate comparisons across interventions, promoting allocative efficiency (3). The use of QALYs is contested in several countries, including the USA (4) and Germany (5), with suggestions that QALYs lack relevance or sufficient sensitivity in some contexts (6). Other health outcomes used include life-years gained, for example, in Denmark, France, and Germany; reduced complications, side-effects, or hospital admissions in Estonia and Latvia (2). Other countries allow a complementary approach by including broader non-health outcomes in certain settings (further details below), including, Canada (7), the Netherlands (8), and England (9). Non-health outcomes might include benefits affecting well-being, or benefits derived from healthcare delivery processes, beyond the care recipient or outside the healthcare sector (10).

Questions of what outcomes to include to promote allocative efficiency and evaluate have been debated by many HTA-related bodies, including the International Society for Pharmacoeconomics and Outcomes Research’s (ISPOR) HTA Council working group (2019) as reflected in their consideration of Good Practices (11), including a “going beyond QALY” approach in HTA. Although it might seem intuitive that health should be the maximand of health services, many have challenged this, including Harris, Mooney, and Rawles (12–14). Mooney noted that an exclusive focus on health could be suboptimal if important non-health benefits of healthcare are omitted (13). Whether systems should seek to maximize health *per se* or a broader measure of welfare continues to be contentious within health economics (15).

In response to earlier criticism of the health-only scope of HTA, it has been suggested that a *going-beyond-health* approach could provide a richer evaluative space that is more reflective of social values (10). Indeed, the evidence presented below on how several countries already do so indicates the relevance of objectives that complement health maximization (7–9).

### Objective

This article considers how HTA is practiced and what its current context implies for adopting a broader *beyond-health* approach. The focus is on the scope of inclusion of broader non-health outcomes rather than a critique of the sensitivity of instruments measuring QALYs. Our analysis uses the example of HTA in Ireland, considering the various HTA institutions’ contexts and practices. Ireland provides an example of a healthcare system with an explicit role for HTA.

We start with an overview of healthcare in Ireland, followed by a survey of agencies employing HTA evidence. As it is unclear at present if there will be consensus supporting a move to a broader *beyond-health* approach, we examine how consistent, coherent, and adaptable Irish HTA practice is to such a shift should it be supported. Our analysis focuses on the analytical perspectives for outcomes and costs, intervention-specific appraisal processes, and the role of stakeholder consultations in determining the value framework, specifically the role of public and patient involvement and engagement (PPIE).

## Irish healthcare

Ireland has a mixed public–private system. Access to tax-funded public services is free for approximately 40 percent of the population based on income, age, and illness-based entitlements, but exceptions include dispensing fees (16). The remaining population can access public services, subject to co-payments. Approximately 40 percent of the population purchases private health insurance (16). Although currently characterized by public–private mix in care provision, Ireland’s current Sláintecare program of reform plans the expansion of universal, single-tier access to public services (17). At present, the public system is legally required to consider the cost-effectiveness of new drugs. Although there is no formal requirement to assess nondrug technologies, HTA is increasingly being applied to such care. The following section examines the principal bodies that consider HTA evidence in Ireland and what appraisal guidelines they follow.

## HTA in Ireland: Who assesses what and how?

Several agencies conduct or review HTA evidence in Ireland. These include the Health Information and Quality Authority (HIQA), the National Centre for Pharmacoeconomics (NCPE), the National Immunisation Advisory Committee (NIAC), the National Clinical Effectiveness Committee (NCEC), the National Screening Advisory Committee (NSAC), and the Institute of Public Health (IPH) (Table 1).

**Table 1.** Agencies that conduct or review HTA evidence in Ireland

Agency	Established (year)	HTA remit	Drug/nondrug	HTA guidelines	EE methods stipulated/referred
Health Information and Quality Authority	2007	To inform national-level health service decisions, including pharmaceuticals, devices, diagnostics, procedures, care pathways and public health	Drug and nondrug	National HTA guidelines, incl. a reference case for economic evaluation (18)	Yes
National Centre for Pharmacoeconomics	1998	To provide HSE reimbursement committees with advice on whether drugs should be funded	Drug	National HTA guidelines, incl. a reference case for EE (18); supplementary guidance on costing (19)	Yes
National Clinical Effectiveness Committee	2010	To oversee the development of clinical guidelines and audit for improving the quality, safety, and cost-effectiveness of healthcare by reviewing existing evidence, including economic evaluation	Nondrug	National HTA guidelines (20)	Yes
National Immunisation Advisory Committee	1998	To provide the Department of Health with advice on vaccines, immunization, and related matters	Nondrug	Unclear	Unclear
National Screening Advisory Committee	2019	To advise the Minister and Department of Health on proposals for introducing and modifying screening programs	Nondrug	Appraisal criteria for screening (21)	No, but referenced CEA
Institute of Public Health	1998	To promote health and well-being and to reduce health inequalities	Nondrug	HIA guidance (22)	No, but referenced EE

CEA, cost-effectiveness analysis; EE, economic evaluation; HTA, health technology assessment; HSE, health service executive; HIA, health impact assessment.

### Health Information and Quality Authority

Under the 2007 Health Act, HIQA has the mandate to undertake HTA to inform national-level health service decisions regarding inventions including pharmaceuticals, devices, diagnostics, procedures, care pathways, and public health. HIQA uses HTA to advise the Minister for Health (henceforth: the Minister) and the Health Service Executive (HSE), Ireland’s state body responsible for delivering public healthcare. HIQA primarily conducts HTA of nondrug technologies and typically does so in response to requests from the Minister or HSE.

## HIQA’s reference case

HIQA published Ireland’s first national HTA guidelines in 2010 and makes regular revisions based in part on public consultation (18). The guidelines state the primary measure of health should be the QALY as derived from EQ-5D (three or five level) or SF-6D instruments and all QALYs should be assumed of equal value. The reference case only considers the HSE’s costs, not those of other government sectors or patients, and thus excludes productivity effects. Although HIQA’s reference case stipulates the HSE’s perspective, it permits a societal perspective in secondary analyses if it is expected to impact results significantly. However, what constitutes a significant impact is unspecified (Table 2).

**Table 2.** Summary of HIQA reference case

Element of health technology assessment	Reference case (HIQA)
Type of economic evaluation	Cost–utility analysis
Outcome measurement	Quality-adjusted life-years (QALYs). The use of generic preference-based methods such as the EQ-5D or SF-6D are recommended to measure utilities
Source of data for measurement of health-related quality of life	Reported directly by patients
Source of preference data for valuation of changes in health-related quality of life	“Informed” general public
Perspective on outcomes	All health benefits accruing to individuals
Perspective on costs	The publicly funded health and social care system in Ireland, that is, Health Service Executive (HSE)
Comparator	Routine care in Ireland
Synthesis of effectiveness	Based on a systematic review
Discount rate	Apply an annual rate of 4% on costs and outcomes occurring after the first year
Equity weighting	Equal weighting should be applied to the outcome measure

Source: (18) Guidelines for the Economic Evaluation of Health Technologies in Ireland.

HIQA’s guidelines do not specify a cost-effectiveness threshold but notes the threshold has varied between EUR20,000 and EUR45,000 per QALY in the past and that both reimbursement within these levels is not guaranteed and technologies above these thresholds have been adopted.

## National Centre for Pharmacoeconomics

Established in 1998, the NCPE is Ireland’s dedicated pharmacoeconomic assessment body. It primarily reviews drug manufacturers’ reimbursement applications and issues recommendations to HSE reimbursement committees on whether drugs should be funded.

The NCPE follows HIQA’s reference case. The cost-effectiveness threshold informing NCPE recommendations has been defined by agreements between the State and a manufacturer representative body. Although past agreements featured a EUR45,000 per QALY threshold, the recent 2021 agreement does not mention thresholds (19).

## Other agencies

*The NCEC* has overseen the development of clinical guidelines and audits for improving the quality, safety, and cost-effectiveness of healthcare since 2010. NCEC guidelines incorporate health economic evidence by systematically searching existing studies and examining their applicability to Ireland. These guidelines cite HIQA’s HTA guidelines (20). Accordingly, although the NCEC’s evidence development is primarily based on review, this process is clearly framed within HIQA’s HTA guidelines. Thus, the choice of outcome measures and perspective are informed by the same HIQA guidelines.

*The NIAC* was established in 1998 to provide the Department of Health with advice on vaccines and related matters. It reviews evidence on new and licensed vaccines and develops immunization guidelines.

Like the NCEC, NIAC uses economic evidence, though it does not make explicit what evidence it appraises and according to what guidelines. Furthermore, NIAC does not appear to systematically publish what cost-effectiveness evidence it considers when forming advice. NIAC has commissioned both the NCPE and HIQA to conduct evaluations of vaccines. Although NIAC is not explicit about the health outcomes assessed or perspective applied, the analyses are consistent with the HTA methods applied elsewhere in Ireland.

*The NSAC* was established in 2019 and advises the Minister and the Department of Health on introducing and modifying screening programs. NSAC issued criteria for appraising screening (21), which explicitly referenced cost-effectiveness, though exactly how it does so is not stated.

*The IPH* was established in 1998 to promote health and well-being and reduce health inequalities. It conducts health impact assessments (HIAs) and has developed HIA guidance, with an explicit reference to economic evaluation (22). Though, like NSAC, it does not make its methods explicit.

Finally, academics and HTA consultancies can undertake analyses for research purposes and on behalf of the healthcare industry. Although analyses vary, best practice is for assessment in accordance with HIQA’s guidelines.

## Summary

Although Ireland might at first appear to have a disparate set of organizations involved in HTA over various intervention types, the processes are more consistent than is initially apparent. Although the HTA framework is not common to all technologies and is not mandatory for nondrug interventions, the HIQA guidelines and its reference case approach are either explicitly or implicitly those primarily adopted in practice by national bodies. Accordingly, Ireland’s cost-effectiveness framework within HTA is relatively cohesive, employs the HSE perspective, and seeks to maximize health as measured in QALYs.

## Prospects for expanding HTA’s scope in Ireland

This section provides further consideration regarding prospects for expanding HTA’s scope of appraisal by drawing on methodological evolution in academic literature and international practice. Our discussion continues under the following subheadings: analytical perspective on outcomes and costs, intervention-specific appraisal processes, and reflection on social values. In the authors’ view, these topics merit further consideration should a decision be made to expand HTA’s scope.

## Analytical perspective on outcomes

Appraisal of intervention outcomes informs allocation decisions regarding optimal care and value for money. What outcomes are considered and how they are measured provides information to support healthcare planning while accurately reflecting service users’ views.

The primary outcome considered within Irish HTA is health, as evidenced by the recommended and widespread use of QALYs. We now examine some relevant international examples to illustrate the variation in HTA practice regarding the scope of outcomes evaluated.

Several countries currently basing reimbursement decisions on QALYs recognize the importance of non-health outcomes. For example, England and Wales’s National Institute for Health and Care Excellence (NICE) state that when the incremental cost-effectiveness ratio (ICER) is between GBP20,000-GBP30,000/QALY (EUR22,600-EUR 34,000; conversion factor 1.13 (23)); NICE will consider “aspects that relate to uncaptured benefits and non-health factors” (9). This is further reflected in NICE’s guidelines for evaluating social care, which recommend using social care-related quality of life, capability, and well-being measures. NICE also allows the use of cost-consequence analysis and considerations of “process characteristics” of healthcare technologies that people value independently of any direct health effects, such as improving convenience (9).

Canadian health economic evaluation guidelines recommend incorporating non-health outcomes, such as reductions in crime or improved educational achievements and suggest a cost-consequences framework to complement cost–utility analysis (7). Notably, the guidelines state that the value of non-health outcomes should be assessed in terms of health trade-offs, suggesting the need to examine society’s willingness to sacrifice health for non-health benefits. Regarding including such non-health outcomes, the literature highlights a possible mismatch between what patients want and what society regards as relevant. For example, compared with patients, taxpayers may place less value on spending that does not directly affect health status, such as improved amenities (10).

The example of non-health benefits within Canadian guidelines usefully highlights questions of whether and how broader consequences could be considered. These include whether a cost-consequence analysis is suitable for trade-offs between health and non-health outcomes and whether a multicriteria decision analysis is suited to formalize such deliberations. Furthermore, we should recognize the distinction between appraising these outcomes and their influence on decision-making. It is one thing to expand the scope of analysis to include other outcomes but it is another to agree explicit weights over these outcomes that then define how they collectively influence a decision to accept or reject an intervention. This illustrates the practical questions posed for implementing broader outcomes within HTA.

The Dutch National Health Care Institute identifies specific considerations for economic evaluations according to the application context, that is, prevention, diagnostics, medical devices and long-term care (8). For example, regarding long-term care, the guidelines state “the objective is not primarily a health benefit in terms of quality of life assessed with an instrument such as the EQ-5D or life expectancy. Instead, goals are set that mainly target “well-being” or “welfare”, such as an improved living environment, living independently for as long as possible, or more social interaction” (8). In this instance, the guidelines recommended a well-being capability measure, ICECAP-O (8).

An oft-cited concern of expanding HTA’s scope is double-counting of outcomes. However, innovative methods are emerging to overcome this concern. For example, Reed *et al.* (24) used a discrete choice experiment to quantify the value of hope. This construct was isolated from gains in quantity and quality of life, thereby avoiding double-counting QALY benefits.

In theory, the QALY framework for measuring and valuing health gains can incorporate non-health outcomes (25). In practice, including non-health outcomes can be challenging. The ideal metric of *benefit beyond health* has a long list of attributes including: being universally applicable to all healthcare interventions and patient groups; sensitive to changes in health and related benefits; cover a wide range of domains of health and related benefits; be appropriately weighted; informed by evidence of patient preferences; potentially suitable for equity weighting all while being supported by consensus within the health economic analysis community. Clearly it is easy to point out generalities of improvement but is hard to design specific tools that meet all these requirements. This suggests it may not be necessary to abandon the QALY framework but rather develop instruments that capture broader outcomes within a QALY-based approach.

HIQA’s guidelines recommend either the EQ-5D or SF-6D QALY instruments. Both are conventional, generic, preference-based instruments to assess health-related quality of life. They do not capture critical social care outcomes that patients and relatives report as important, including independence, confidence, safety, overall well-being, capability, or empowerment (26). Both exclude healthcare process-related aspects, such as being treated with dignity and at a convenient time and location. HIQA’s guidelines signal an awareness of the limitations of the EQ-5D and SF-6D and a willingness to consider disease-specific instruments. Although disease-specific instruments may not necessarily capture benefits *beyond health* (nor inform allocative efficiency), this acknowledgement at least shows readiness to consider other instruments.

Preference-based instruments that capture broader benefits include the Adult Social Care Outcomes Toolkit (ASCOT), EQ Health and Wellbeing (EQ-HWB), ASCOT-Carer for social care or CarerQol-7D for carers, and well-being capability measures (ICECAPs, OxCAP-MH). Growing evidence supports the application of these broader instruments internationally and in Ireland.

## Analytical perspective on costs

Critics of the payer perspective note that it likely does not capture all items of social value and thus may not optimize welfare (27). Evidence suggests a broader societal perspective matters for the complete appraisal of health technologies where morbidity and long absences from work are probable; for social care services, where informal carers provide the support that may impact mainstream statutory services through reductions in hospitalizations, for example, in cancer, mental health, or neurodegenerative conditions.

Consideration of informal care can have a significant impact on the cost-effectiveness of certain technologies. Lin *et al.* (28) found in 85 percent of analyses, including Alzheimer’s disease/dementia spillover costs or health effects, reduced ICERs or kept the intervention cost saving. Accordingly, the societal value of such interventions may be underestimated if spillover effects are excluded. Several experts recommend economic evaluations of social care, in general, adopt multiple perspectives when examining intervention costs (29).

Irish HTA primarily adapts the “narrow” HSE perspective as reflected by HIQA’s reference case. However, as noted above, it allows for the societal perspective as a secondary analysis. Analyses from a range of perspectives could assist in identifying cost-shifting between sectors and possible perverse incentives. If technology is cost-effective from a public sector perspective but shifts costs to service users and/or unpaid carers, this cost shift should be clearly stated alongside the study’s conclusions. Cost-shifting can be an important issue in a system such as Ireland’s, where the health system is not fully universal, and some patients are exposed to co-pays and is particularly relevant in appraisals of social care and public health interventions.

## Intervention-specific appraisal processes

In Ireland, the formal HTA process currently primarily applies to drugs. Although such evaluation is important and all aspects of value that matter to patients should be accounted for, those same principles should be applied across the spectrum of health and social care services.

Several countries have parallel systems for appraising social care, public health and other nondrug technologies and are potentially informative for Ireland. For example, NICE sets context-specific appraisal guidelines such as Social Care Guidance and Public Health Guidance. Canada facilitates nondrug technologies appraisal by generating HTA information within individual provinces consistent with the Canadian Agency for Drugs and Technologies in Health (CADTH) guidelines (30). Several hospitals have established HTA units to support local decision-making, as by law, all academic medical centers must have their own HTA capacity. The Netherlands developed specific considerations for economic evaluations in prevention, diagnostic, medical devices and long-term care (8).

In Ireland, the most coherent form of HTA relates to drugs through the NCPE. HTA of nondrug technologies, social care and public health interventions is not mandatory and is distributed across other bodies. Although these appraisals are largely made according to HIQA’s guidelines, there is a question of the frequency, volume, and impact of these appraisals (31).

A major contributor to the relative lack of focus on HTA for nondrug technologies is the absence of suitable data. Most services and procedures do not have readily available data on intervention effectiveness and often are captured by administrative data. Cost data are also not as readily available, and there is often substantial price variation (32). One way to facilitate HTA of nondrug technologies is through real-world data. These can capture information on costs and health outcomes that matter to patients and caregivers, thereby adding value to existing information in limited administrative claims. Greater use of data sources outside of clinical trials opens more possibilities in ways that align with patients’ preferences and recognizes how outcomes important to patients can vary across disease areas and individuals.

Whether intervention-specific appraisal processes, such as tailored assessment frameworks for particular classes of intervention such as screening, vaccination, medical devices or public health interventions, are beneficial is unclear. They could permit justified nuance of HTA in particular contexts, thereby enhancing decision-making sensitivity to important considerations. Conversely, they may create unjustified divergence of HTA methods that compromise comparability of health economic evidence between intervention classes. The alternative to intervention-specific appraisal processes is the expansion of a common reference case process that is applied to all interventions. The concomitant respective benefits and challenges of a unified approach are the comparability and potential insensitivity of appraisals. Further examination of the value of intervention-specific appraisal processes appears warranted.

## Patient and public involvement and engagement & social value

Engaging patient groups and the public in appraisal processes may better inform the inclusion of broader outcomes and social values in HTA. The rationale for such an engagement has been widely documented (33). It would delineate a role for the patient as an “expert witness” with unique insight into living with an illness and the potential benefits/disadvantages that interventions offer. Such patient insights may be particularly helpful in addressing discrimination concerns that can arise with the application of health-related quality of life weighting systems. For example, does accounting for disability imply lower baseline quality of life and result in a biased valuation of interventions for such populations. Such involvement may assist by better reflecting what is truly valued.

Despite the recognized value of patient and public views, there remains a lack of systematic and explicit incorporation in HTA. In response, efforts have been initiated to facilitate PPIE and the reflection of social values (33). NICE has been at the forefront, using a standing Citizens’ Council, to inform perspectives on ethical issues in healthcare policy. Recommendations from a Citizens’ Council indirectly inform NICE’s guidance through their inclusion in the NICE “Social Value Judgements” document (34). Several frameworks for PPIE have been proposed since then, including in Australia (35), Canada (36), Malaysia (37), and Ireland (38). Despite the growing effort to facilitate PPIE, there remain fundamental questions about the extent, quality, and impact of these frameworks.

From a methodological perspective, patient and public values and preferences for health technologies could be incorporated into HTA through several approaches, including quantifying the domains and harmonizing them into model inputs (39). Stated preferences methods used to study preferences and values for healthcare services, treatments, and outcomes are well-suited to the task, which is essential for translation to HTA.

Ireland has taken steps to incorporate PPIE, and its value is recognized. This is partly demonstrated by the Health Research Board’s Ignite PPI initiative. Similarly, HIQA’s guidelines are open to regular review and consultation. However, it is crucial to ensure the diversity of population views, values and circumstances are involved systematically and explicitly in discussions and decisions. For example, evidence suggests that people living with dementia rarely have a say in healthcare planning or the agendas and priorities of the research projects that aim to help them. PPIE in HTA requires awareness of who is currently excluded from decision processes and a willingness to listen to and incorporate diverse priorities and preferences. Critically, PPIE is not a once-off consultation but an ongoing process. Formal inclusion of diverse population groups would further allow the inclusion of broader benefits and social values within the Irish HTA. A clear definition of PPIE and its function in HTA with a deliberative process, such as citizen panels, is one potential approach to facilitate the inclusion of broader benefits and improvement of the quality of measurement within HTA.

### Summary

Overall, Ireland has established a relatively cohesive practice for generating HTA evidence by establishing national HTA guidelines. The adoption of a single set of national guidelines that informs HTA practice across all institutions ensures consistency. HIQA’s guidelines and Irish HTA practice more generally primarily adopt the health maximization approach, though it allows for alternative, validated, disease-specific quality of life instruments in cases where the recommended EQ-5D or SF-6D may not be sufficiently sensitive to capture what may be considered a clinically meaningful change in health status. Additionally, the use of a cost-effectiveness threshold range could be considered as soft evidence that the system already considers aspects beyond strict health gains.

HIQA regularly consults on its guidance, which offers an avenue for reform that is open to all. This is an important and healthy part of the process and deserves recognition. The international examples of expanding HTA’s scope of outcomes indicate the important operational considerations regarding how they inform decision-making, many of which are not trivial.

## Concluding remarks

We surveyed the Irish HTA framework to examine how economic evidence is evaluated, such as who assesses what and how. This informed our consideration of how consistent, coherent, and adaptable HTA is in Ireland to prospects for an expanded scope of appraisal toward the *beyond-health* approach.

Our analysis indicates Ireland’s current HTA guidelines largely show receptiveness for a “broader” evaluative space. Ideally, economic evaluation within HTA will involve enhanced PPI engagement, and the set of benefits recognized by Irish HTA guidelines would reflect the nation’s societal preferences. The differences in context-specific appraisal should reflect genuine differences in preferences rather than accidents of history or administrative inertia. As Irish HTA matures, these questions will naturally emerge. It is maybe the time for Ireland to reflect on these. Although our focus is on Irish HTA processes, the discussion applies more generally to other countries’ use of HTA.

As the next step, we recommend a multi-stakeholder working group that brings together leading experts from a wide range of disciplines and stakeholder perspectives to recommend concrete steps to address enablers and barriers to the *beyond-health* approach. Essential elements of the panel’s work will be to advance an agenda for HTA of nondrug technologies and to apply patient-centered methods to economic evaluations to drive social value and efficiency. We call on our colleagues in Ireland and internationally to engage on this topic as a matter of priority.
